# Plasmid DNA Delivery into the Skin via Electroporation with a Depot-Type Electrode

**DOI:** 10.3390/pharmaceutics16091219

**Published:** 2024-09-18

**Authors:** Yuya Yoshida, Manami Aoki, Kalin Nagase, Koichi Marubashi, Hiroyuki Kojima, Shoko Itakura, Syuuhei Komatsu, Kenji Sugibayashi, Hiroaki Todo

**Affiliations:** 1 Faculty of Pharmacy and Pharmaceutical Sciences, Josai University, 1-1 Keyakidai, Sakado 350-0295, Saitama, Japan; 2 Pharmaceutical Research and Technology Labs., Astellas Pharma Inc., 180 Ozumi, Yaizu 425-0072, Shizuoka, Japan; 3 Laboratory of Biopharmaceutics, Faculty of Pharmaceutical Sciences, Tokyo University of Science, 2641 Yamazaki, Noda 278-8510, Chiba, Japan; 4 Faculty of Pharmaceutical Sciences, Josai International University, 1 Gumyo, Togane 283-8555, Chiba, Japan

**Keywords:** DNA vaccine, DNA delivery, electroporation, skin, dermal

## Abstract

**Objectives:** Non-viral mediated plasmid DNA transfection by electroporation (EP) is an established method for gene transfection. In this study, the usefulness of direct EP at an intradermal (*i.d.*) site (*D*_EP_) with implanted electrodes to achieve a high protein expression level was investigated. In addition, *D*_EP_ application with various intervals with a low application voltage was also evaluated to confirm its effect on protein expression. **Methods**: Green fluorescent protein (GFP)- and luciferase-encoding DNA were administrated, and GFP and luciferase were evaluated. **Results**: A higher protein expression level was observed after green fluorescent protein (GFP)- and luciferase-encoding DNA were delivered by *i.d.* injection followed by *D*_EP_ application. When luciferase expression was observed with an in vivo imaging system, continuous expression was confirmed over 21 days after *i.d.* injection followed by *D*_EP_ at 100 V. This approach provided increased gene expression levels compared with conventional EP methods via the stratum corneum layer. In addition, the effect of application voltage on luciferase expression was investigated; two-time applications (repeated *D*_EP_) at 20 V with 5 min intervals showed similar luciferase expression level to single *D*_EP_ application with 100 V. Histological observations showed the skin became thicker after a single *D*_EP_ at 100 V, whereas no apparent thickness changes were confirmed after repeated *D*_EP_ at 20 V with 5 min intervals. **Conclusions**: This study revealed that direct *i.d.* voltage application achieved high protein expression levels even at low voltages. Skin is a promising administration site for DNA vaccines, so this approach may be effective for DNA vaccine delivery into skin tissue.

## 1. Introduction

The first report on gene transfer into in vitro-cultured cells by the application of electroporation (EP) was made in 1982, and since then EP has been applied extensively for the transfer of DNA into target cells in biotechnological and biomedical applications [1,2]. EP application induces normal fluctuations in the membrane that are magnified by the transmembrane voltage difference induced by the electrical pulse, resulting in large hydrophobic pores [3]. The formation of these pores is reversible, and they facilitate the initial interaction between DNA and the cell membrane. Recent studies showed that plasmid DNA (pDNA) aggregates at the membrane and is primarily internalized via endocytic pathways rather than directly through the pores [4]. Skin is an attractive EP application site because of its accessibility. EP-mediated transdermal delivery was reported by Prausnitz et al. in 1993, thereby demonstrating the potential of this approach [5]. Furthermore, it has been reported that EP application with a high voltage mediated the delivery of macromolecules into the viable skin layer by temporarily disrupting the barrier function of the stratum corneum (SC), the outermost layer of the skin [6,7]. Dujardin et al. reported successful delivery of plasmid DNA (4.7 kb) using EP [6], Zhang et al. demonstrated the delivery of microspheres (2–20 μm) loaded with leuprolide acetate [7].

Vandermeulen et al. [8] studied the effect of administration sites on the immune response after DNA vaccine application followed by EP application, and different immune responses were obtained from different administration sites. They also reported that EP application on the skin surface after intradermal (*i*.*d*.) injection of a DNA vaccine induced cellular immunity. Eriksson et al. [9] found that *i*.*d*. immunization with a DNA vaccine assisted with EP was less invasive and caused less patient discomfort.

During EP treatment, because the electrical resistance of the SC layer is higher than that of deeper tissues due to composition differences between the SC and deeper layers, the high electric field strength resulting from EP application on the skin surface is present mostly in the SC layer. Therefore, cell membranes in deeper skin layers are permeabilized when the threshold transmembrane voltage is reached by application of a high voltage on the skin surface [10,11].

Many researchers in recent years have reported improved methods to obtain high gene expression levels using various EP types, such as needle-puncture [12], plate-electrode [13,14], and multiple head [15,16] electrodes after injection of pDNA into *i.d.* or muscle tissue. Recently, the development of microelectromechanical systems has led to the emergence of embedded microelectrodes, with a significant advancement in the development of implantable electrodes for internal use. These devices have achieved miniaturization, and in addition to being used as biomonitoring sensors, these implantable devices are also expected to be used for drug delivery systems [17,18,19,20].

Consequently, in the present study, the usefulness of EP with embedded electrodes (depot EP) was investigated after *i.d.* administration of luciferase-encoding pDNA solution by comparing conventional EP electrodes placed on the surface of the SC EP was performed using electrodes placed on the exposed dermis through a skin incision. The skin was then sutured, and this method was assumed to be EP with an embedded electrode to evaluate its effect on luciferase expression.

## 2. Materials and Methods

### 2.1. Materials

A hollow-type microneedle (hMN) for *i*.*d*. injection was purchased from NanoPass Technologies Ltd. (Micron-Jet^TM^, Nes Ziona, Israel). The hMN had three pyramid-shaped microneedles (MNs) of 0.6 mm length. MicronJet MNs are made of silicon with very sharp tips that penetrate the epidermis followed by drug delivery through three channels. Injection with the hMN was conducted by insertion of the needle at an approximately 45° angle and kept the same position during administration. The 27 G injection needles were purchased from Terumo Co. (Tokyo, Japan). The EP system was provided by BTX (Holliston, MA, USA). All reagents were of analytical grade and were used as received without further purification. Luciferase-pcDNA3 was a gift from William Kaelin (Addgene plasmid #18964; http://n2t.net/addgene:18964/ (accessed on 12 September 2024); RRi.d.:Addgene_18964). pcDNA3-EGFP was a gift from Doug Golenbock (Addgene plasmid #13031; https://www.addgene.org/13031/ (accessed on 12 September 2024; RRi.d.:Addgene_13031).

#### 2.1.1. Preparation of pDNA

pDNA (pcDNA3-luciferase, pcDNA3-EGFP) was transformed into *E. coli* DH5α (TOYOBO, Osaka, Japan) as host cells by heat shock at 42 °C. The amplified pDNA was purified using a QIAGEN Plasmid Giga kit (QIAGEN, Hilden, Germany) in accordance with the manufacturer’s instructions. pcDNA3-luciferase (0.2 mg/mL) and pcDNA3-EGFP (4 mg/mL) were dissolved in phosphate-buffered saline (PBS, pH 7.4) for administration. The recombinant DNA experiments were approved by Josai University Biosafety Committee for Recombinant DNA Research (approval number JU2019-9).

#### 2.1.2. Animals

Male BALB/cCrSlc mice (weight 20 g, 6 weeks old) were purchased from Sankyo Labo Service Corporation, Inc. (Tokyo, Japan). Male BALB/cCrSlc mice were kept in a room regulated at 25 ± 2 °C with a light/dark cycle (on and off time: 9:00–21:00) every 12 h. Water and food (MF, Oriental Yeast Industry, Tokyo, Japan) were freely available. All experiments were performed in accordance with the ARRIVE guidelines (https://arriveguidelines.org accessed on 12 September 2024) and the Guidelines for the Institutional Animal Care and Use Committee of Josai University. The animal experiments were approved by the Institutional Animal Care and Use Committee of Josai University (Sakado, Saitama, Japan). After approval by the Josai University Ethics Committee (approved numbers JU22005 and JU23004), the experimental animals were used in accordance with the Josai University Laboratory Animal Regulations.

#### 2.1.3. EP Device Preparation

**Figure 1:**
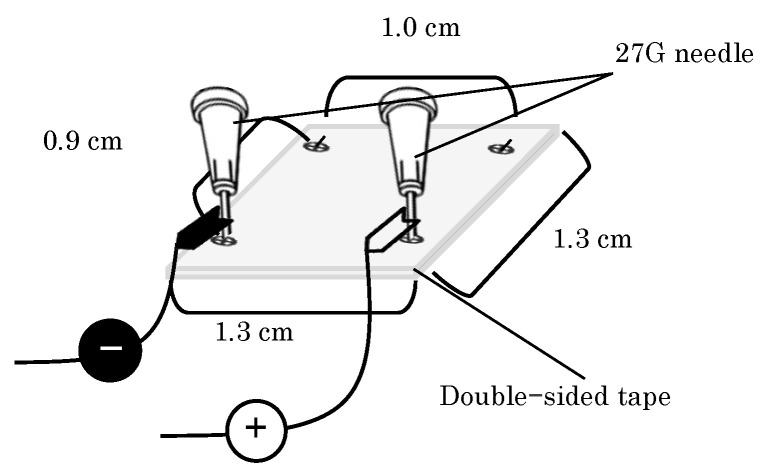
Schematic diagram of the fabricated fixation type EP device made from 27 G needles and rubber.

The fixation-type electrode used in the present study for EP application is shown in Figure 1. The electrodes were fabricated by bending 27 G needles and fixing them with double-sided tape so that the electrode length in contact with the skin was 0.9 cm.

### 2.2. Methods

#### 2.2.1. Observation of GFP Expression in Mice Abdominal Skin Using a Laser Scanning Microscope

Mice were anesthetized with triple anesthesia (intraperitoneal administration of 0.2 mg/kg medetomidine hydrochloride, 2.5 mg/kg butorphanol, and 2 mg/kg midazolam tartrate). Then, 20 µL of EGFP-pDNA at a concentration of 0.2 mg/mL prepared with PBS (pH 7.4) was delivered by *i.d.* injection using an hMN. After *i.d.* injection, the abdominal skin of the mice was incised, and a fixation-type electrode was placed on the dermis exposed through the incision. The fixation-type electrode was fixed in position with double-sided tape. The anode and cathode in the fixation-type electrode were then connected to the EP system for voltage application. EP was applied using a squared wave pulse generator, 99 msec for ten pulses were applied at 100 V. During EP, the fixation-type electrode was kept on the dermis surface. As a comparison, EP application through fixation-type electrodes placed on the SC was also performed. To avoid surgical effects, all studies were subjected to the same surgical treatment as the EP application from the dermis side shown above. In the present study, EP application with the fixation-type electrode from the dermis and SC sides indicate direct EP at an *i.d.* site (*D*_EP_) and conventional SC EP (*C*_EP_), respectively.

After *i.d.* injection of pDNA solution a 10 mm wide skin region was excised at 24 h after the EP application, and the skin tissue was embedded using the Kawamoto method [21] to prepare frozen blocks. The frozen blocks were cut into skin cross-sections with 10 μm thickness using a cryostat (CM3050S; Leica, Wetzlar, Germany) to view the GFP expression area between the cathode and anode.

After obtaining tissue sections, GFP expression in the tissue was observed using a confocal laser scanning microscope (CLSM) with a wavelength of 473 nm (Fluoview FV3000 and software: FV31S-SW version 2.6.1.243, Olympus, Tokyo, Japan).

#### 2.2.2. In Vivo Imaging System Observation

In addition to GFP expression in tissues, expression of luciferase was observed for 21 days (observation period: 4, 10, 24 (1 day), 30, 48 (2 days), 96 (4 days), 168 (7 days), 336 (14 days), 504 (21 days) h after luciferase-encoding pDNA, Luc-pDNA, administration). The same conditions for EP application were applied for *D*_EP_ and *C*_EP_ with fixation-type electrodes. In addition, the effect of an application voltage of 20 V and two EP applications with 5 or 10 min intervals (repeated EP) was investigated. The repeated EP conditions of the number of electric pulses and the duration were the same as for application conditions with 100 V (10 pulses, 99 msec duration).

Luciferase expression was measured using an in vivo imaging system (IVIS^®^ Spectrum) (PerkinElmer^®^ Ltd., Waltham, MA, USA). Mice were anesthetized with 1.5–2.0% isoflurane inhalation anesthesia, and 100 µL of VivoGlo^TM^ Luciferin, In Vivo Grade (Promega, Madison, WI, USA) solution with PBS at a concentration of 30 mg/mL was delivered by subcutaneous injection using a 27 G needle at 150 mg/kg. After 15 min, luminescent images of the whole body were acquired using the IVIS Spectrum, and the luminescence intensity in each region of interest (ROI) was quantified using Living Image 4.7.3 software (PerkinElmer, Waltham, MA, USA).

#### 2.2.3. Quantification Analysis of Luciferase Expression

Protein quantification was conducted 24 h after Luc-pDNA administered and EP application. The skin was excised as described above (Section 2.2.1), the skin was homogenized using CRYO PRESS (MICROTEC CO., LTD., Chiba, Japan), then suspended in Lysis buffer (Luciferase Cell Culture Lysis 5X Reagent, Promega, Tokyo, Japan) and ultrasonic homogenizer VCX-750 for 1 min (SONICS & MATERIALS, INC., Newtown, CT, USA). After homogenization, all samples were centrifuged (21,200× *g*, 4 °C, 5 min) and the supernatant was collected. Luciferase activity in the extracted protein samples was measured using a plate reader (Synergy H1, Agilent Technologies, Santa Clara, CA, USA). Light emission was measured for 10 s, and the results were expressed as relative light units (RLU). Luciferase activity was expressed as RLU per mg of total protein (RLU/mg protein). This was calculated by dividing the RLU value obtained from the luciferase assay by the total protein concentration determined using BCA (FUJIFILM Wako Pure Chemical) assay. In addition, non-EP-treated skin was used as a control.

#### 2.2.4. Thickness of the Skin

Skin samples after EP application were excised and embedded using the Kawamoto method and stored at −80 °C, and continuous skin sections were prepared at 10 µm thickness using a cryostat (CM3050S; Leica, Wetzlar, Germany). The prepared skin sections were then stained with hematoxylin and eosin (H.E.) for observation using a microscope (BZ-X710; KEYENCE, Osaka, Japan), and the thickness of skin sections was measured using a BZ-X Analyzer (version 1.4.1.1, KEYENCE). Five representative sections, evenly distributed throughout the excised skin sample and without any obvious damage, were selected from each specimen for measurement. For each selected section, the whole skin thickness was measured, and the average of these five measurements was used as the representative thickness for that specimen. All skin samples used in the histological observations were from skin incised with or without EP application.

#### 2.2.5. Statistical Analysis

Statistical analysis was performed with JMP^®^ Pro (ver. 15.0.0, SAS Institute Inc., Cary, NC, USA). The statistical significance of differences was examined using one-way analysis of variance (ANOVA) followed by a Tukey–Kramer post-hoc test. The significance level was set at *p* < 0.05.

## 3. Results

### 3.1. GFP Expression in the Skin after i.d. Administration Followed by EP Application

**Figure 2:**
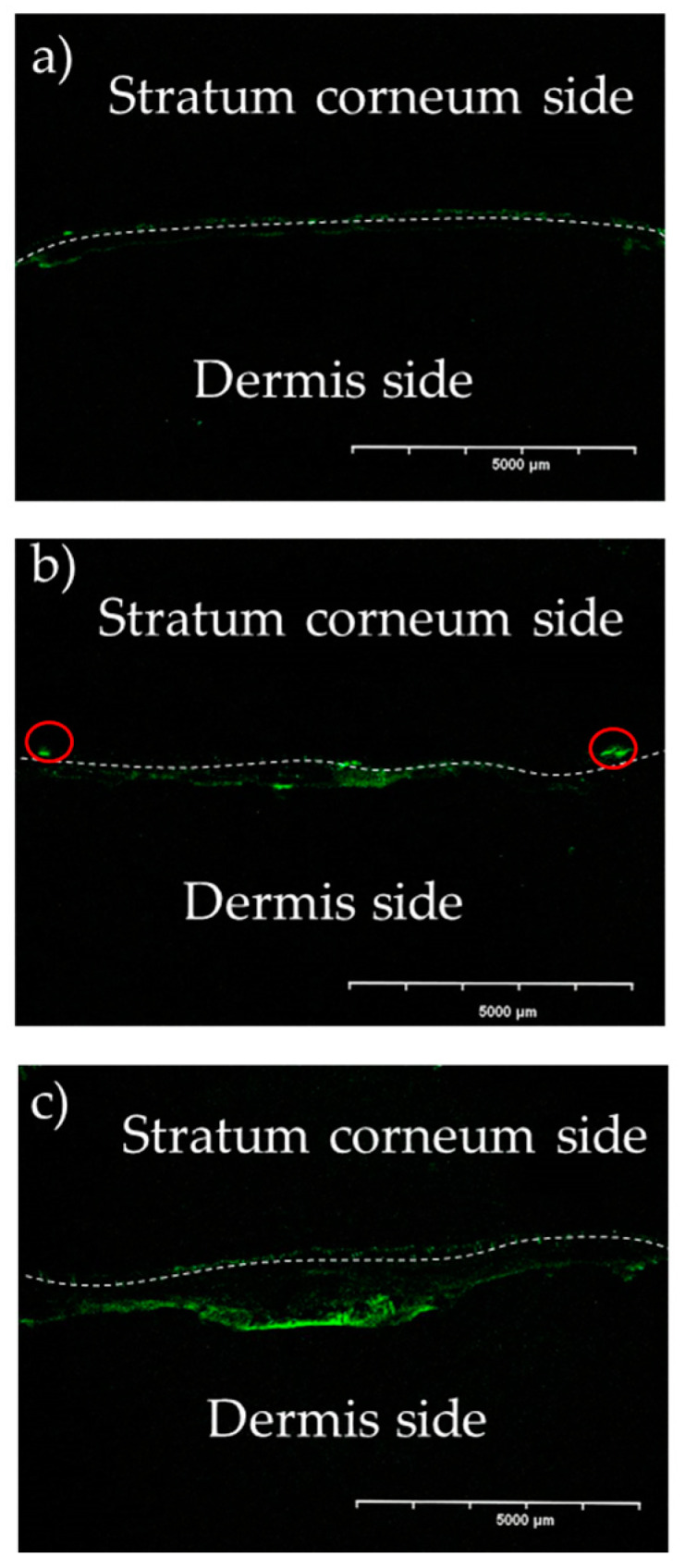
Observation of intradermal GFP expression with or without EP application. The dotted line indicates the interface between the SC and the viable epidermis. (**a**) *i.d.* injection of GFP-encoding DNA (*i.d.* only, without EP application), (**b**) *C*_EP_ after *i.d.* injection of GFP-encoding DNA, and (**c**) *D*_EP_ after *i.d.* injection of GFP-encoding DNA.

Figure 2 shows the skin distribution of GFP expression in skin samples from mice at 24 h after *i.d.* injection of the EGFP-encoding pDNA solution followed by EP application with a fixation-type electrode. The *i.d.* distribution of fluorescence derived from GFP expression was observed with histological analysis of skin vertical sections using a CLSM. When the EGFP-encoding pDNA solution was delivered by *i.d.* administration without EP application (*i.d.* only), no significant fluorescence derived from GFP expression was observed at the boundary surface between the SC and viable epidermis layers. On the other hand, *D*_EP_ and *C*_EP_ were conducted, and distinct green fluorescence was confirmed in the skin vertical section. Notably, GFP expression was localized at a shallow depth in the skin when *C*_EP_ was applied, whereas green fluorescence was observed at greater depth in the skin when *D*_EP_ was applied. In addition, higher fluorescence was confirmed in the application of *D*_EP_ compared with the application of *C*_EP_.

### 3.2. Luciferase Expression in the Skin after i.d. Administration Followed by EP Application

**Figure 3:**
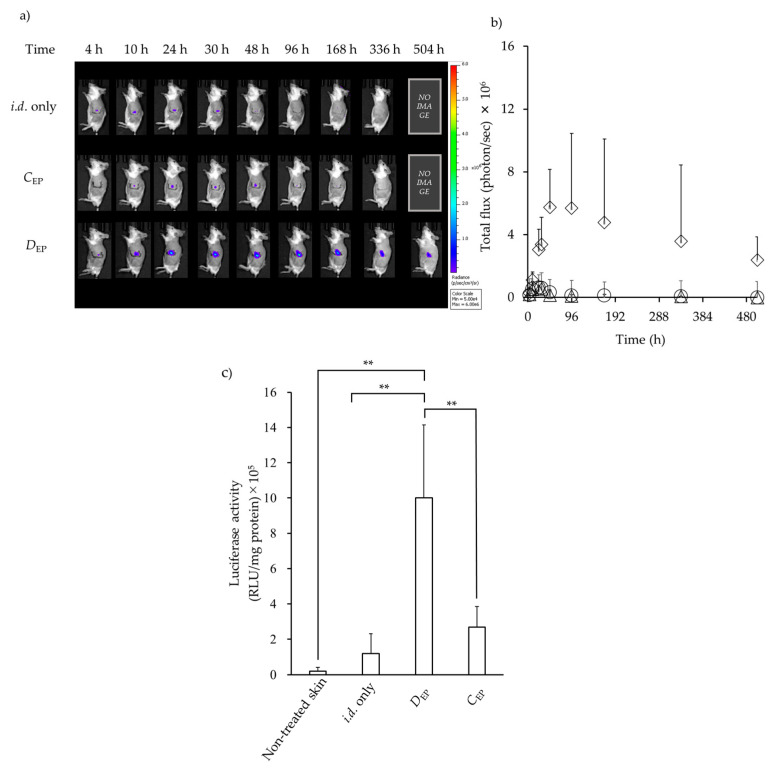
In vivo expression of luciferase after with or without EP application after *i.d.* injection of Luc-pDNA. (**a**) Representative from an in vivo image system (IVIS) of luminescence shown at the administration site of mice at different time points after *i.d.* injection with or without EP. (**b**) Time course of luciferase expression throughout 21 days was performed with the total luminescence (photons/sec) with the same size of ROI. Symbols: △; *i.d.* only, ○; *C*_EP_ after *i.d.* injection of Luc-encoding DNA, ◊; *D*_EP_ after *i.d.* injection of Luc-pDNA. (**c**) Quantitative analysis expression of luciferase 24 h after *i.d.* injection. Bars represent different treatment groups: non-treated skin, *i.d*. only, *D*_EP_, and *C*_EP_. Data are represented as mean + S.D. (*n* = 3–5). ** *p* < 0.001.

Figure 3 shows the time course of luciferase expression following *i.d.* administration of Luc-pDNA solution, followed by EP application with *D*_EP_ or *C*_EP_. As a comparison, *i.d.* injection without EP (*i.d.* only) was also evaluated. Luciferase signal at the *i.d*. injection site of Luc-pDNA solution was confirmed in all experiments using IVIS (Figure 3a). The luciferase signals were compared by gating the individual total flux (photons/seconds) (Figure 3b). At 10 h after *i.d.* injection, the maximum photon flux that indicated the expression levels reached for *C*_EP_ and without EP application (*i.d*. only). On the other hand, the maximum photon flux was reached at 48 h for *D*_EP_. The luciferase expression levels after *D*_EP_ were much higher than those after *C*_EP_ and without EP. The expression levels were in the order *D*_EP_ > *C*_EP_ > *i.d*. only. The expression levels were significantly different among the three conditions (*p* < 0.001). *D*_EP_ resulted in significantly higher expression than *C*_EP_ (*p* < 0.001), and *C*_EP_ showed significantly higher expression than *i.d*. only (*p* < 0.001). In addition, the photon flux values showed a gradual decrease, but higher luciferase expression continued even at 21 days (504 h) after *D*_EP_ application.

Figure 3c shows the quantitative analysis of luciferase expression over 21 days after *i.d.*, administration with or without EP application. Weak luciferase expression was confirmed after *C*_EP_ was applied. On the other hand, *D*_EP_ resulted in higher luciferase expression compared with other application methods, which was in good agreement with the results of luciferase expression in Figure 3b. *D*_EP_ generated approximately 10-fold and 5-fold more efficient luciferase expression than *i.d.* only and *C*_EP_ administration, respectively, when luciferase expression was compared 24 h after *i.d.* injection of Luc-pDNA.

### 3.3. Luciferase Expression in the Skin after i.d. Administration Followed by EP Application

**Figure 4:**
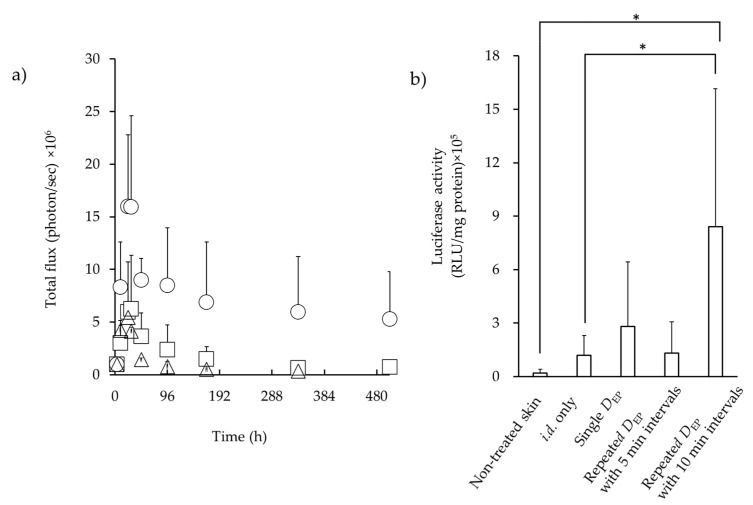
In vivo expression of luciferase with repeated EP application after *i.d.* injection of Luc-pDNA. Representative luminescence image obtained from an in vivo image system (IVIS) at the administration site in mice at different time points after *i.d.* injection. (**a**) Time course of luciferase expression throughout 21 days was performed for total luminescence (photons/sec) for the same size of ROI. Symbols: △; single *D*_EP_, ☐; repeated *D*_EP_ with 5 min intervals, ○; repeated *D*_EP_ with 10 min interval. (**b**) Quantitative analysis expression of luciferase 24 h after *i.d.* injection. Data are represented as mean + S.D. (*n* = 3–5). * *p* < 0.001.

*D*_EP_ at an application voltage of 100 V exhibited enhanced luciferase expression compared with *i.d.* only and *C*_EP_ administration; thus, the effects of repeated EP application with a low voltage on luciferase expression were investigated with *D*_EP_. Figure 4a shows the time course of luciferase expression after *i.d.* administration of Luc-pDNA solution, followed by EP application with *D*_EP_ at an application voltage of 20 V with varying intervals between application times. After a single application of *D*_EP_ at a 20 V, weak luciferase expression was observed. On the other hand, repeated *D*_EP_ application at 20 V showed increased luciferase expression, and especially using a 5 min interval, exhibited higher expression compared with a single application of *D*_EP_ at a 20 V. However, luciferase expression after repeated *D*_EP_ application at 20 V with a 10 min interval exhibited a similar expression profile as a single *D*_EP_ application at 20 V. Figure 4b shows the effect of repeated *D*_EP_ application on quantitative luciferase expression. A higher level of luciferase expression was observed with repeated *D*_EP_ application at 5 min intervals compared with a single *D*_EP_ application or without EP application, and significant differences were observed only with no EP application.

### 3.4. Skin Thickness Observation after EP Applications

**Figure 5:**
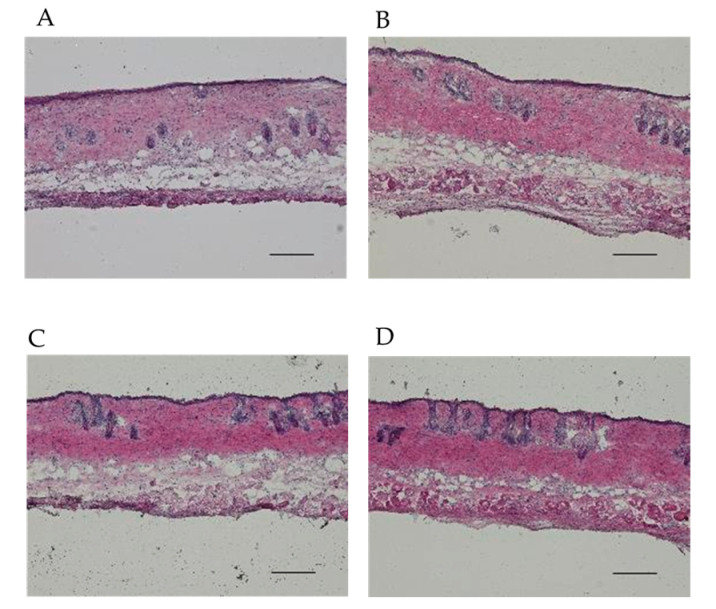
Histological observation of the skin. Photos: (**A**) non-treated skin; (**B**) single *D*_EP_ application at 100 V; (**C**) single *D*_EP_ applications at 20 V; (**D**) repeated *D*_EP_ applications at 20 V at 5 min intervals. Bars indicate 100 µm (vertical slice).

Figure 5 shows histological observations of the skin that consisting of SC and viable epidermis and dermis layers after EP application. The thickness of the skin after *D*_EP_ application at 100 V (694 ± 40.6 μm) was thicker than that of non-EP applied skin (528 ± 9.70 μm). The thicker dermis layer was confirmed in *D*_EP_ application at 100 V. On the other hand, the skin thicknesses after single *D*_EP_ at 20 V (535 ± 23.7 μm) and repeated *D*_EP_ at 20 V (538 ± 12.8 μm) were of similar thickness to the control skin, without EP application.

## 4. Discussion

When tissue is exposed to an electric field in conditions that increase cell permeability, macromolecules are able to pass through the cell membrane. This procedure has been utilized for pDNA delivery [22,23]. EP-mediated in vivo gene delivery has proven highly effective for the treatment of diseases, such as inherited monogenic diseases, cancer, and viral infections. In principle, the application of a high-voltage electric field caused temporary depolarization of a cell membrane and the formation of pores, which allows the delivery of DNA [22]. In addition to the amplitude of the voltage, the frequency number of pulses, pulse shapes (exponential decay, square, and bell shaped), the pulse duration, and distance from the electrode (electric current density) are factors that affect the efficacy of DNA delivery [24].

Skin is an easily accessible tissue and therefore a potential target for gene therapy. The skin has a large surface area, approximately 20,000 cm^2^ in the average human adult, making it a suitable target for DNA delivery. Moreover, Langerhans cells are antigen-presenting dendritic cells that reside in the skin, making them an excellent target for vaccine production [25]. However, *i.d.* administration of DNA using EP involves high-voltage EP loading on the skin surface after DNA undergoes *i.d.* injection. The development of technology consisting of micro-electro-mechanical systems coupled with advances in materials science has enabled the production of small bioelectronics devices, allowing biomonitoring and therapy with implantable devices. The SC plays a crucial role in the overall electrical resistance of skin [26,27,28,29], so that a high EP voltage should be necessary to achieve enough electrical field to deliver macromolecules into cells when EP is applied on the skin surface. Pavselj et al. reported that a high electric field was observed in the SC, whereas the electric field in the deeper layer was below the permeabilization threshold [30,31]. When 400 V/cm or higher was applied, the epidermis and dermis, as predicted by their model, were subjected to an electric field above the threshold. Thus, an implanted EP device in the tissue has the potential to deliver DNA into cells with a low voltage because the voltage may be loaded by avoiding the SC, which has a high electric resistance value. It also enables EP applications that are difficult to perform with puncture-type EP, such as repetitive EP applications. In the present study, the possibility of implanted-type (depot-type) EP application was investigated by comparing protein expression after *i.d.* administration of pDNA with conventional and insertion-type EP.

When *D*_EP_ was applied, GFP expression, shown as green fluorescence, was observed in the dermis layer over a wide area, whereas when *C*_EP_ was applied, it was observed at a shallow depth in the epidermal layer and over a narrow area compared with *D*_EP_. The strength of electric field provided by EP application was not investigated, but a higher expression level was confirmed at the site close to the EP application point, suggesting that DNA was delivered to the area where sufficient improvement in permeabilization was obtained. In addition, a higher luciferase expression level was confirmed even 48 h after *D*_EP_ application (Figure 3 and Figure 4). In contrast, although the luciferase activity determined using skin homogenates after *C*_EP_ was slightly higher than that obtained without EP application, namely *i.d*. administration of pDNA alone (Figure 4), the luciferase expression level obtained after *C*_EP_ application was significantly lower compared with *D*_EP_ for any measurement periods (Figure 3). 

Hirao et al. [32] reported DNA delivery with a subcutaneous EP device with a penetration depth of 3 mm. Subcutaneous EP resulted in higher cellular and humoral responses to a human immunodeficiency virus vaccine in nonhuman primates compared with subcutaneous injection alone. Broderick et al. [33] demonstrated DNA delivery with an *i.d.* EP device at a lower voltage (15 V), and they reported a strong humoral response and the titers developed with a minimally invasive device. Notably, they reported that GFP transfection at 15 V appeared more reproducible and robust with a 50–100 V application with the same device. In the present study, *D*_EP_ with 20 V application at 5 min intervals showed significantly higher luciferase expression compared with that obtained using *i.d.* application alone, although *D*_EP_ with 20 V application at 5 min intervals exhibited a similar luciferase expression to single *D*_EP_ application at 20 V. In the case of macromolecules such as DNA delivery into the cytoplasm, the presence of macromolecules is necessary during electric pulse application. Many reports have been published on DNA transfer in cells [32,34]. Increasing permeation though the membrane by membrane destabilization during EP application is the simplest method [35,36]. Another possibility is that electric fields affect the passage of the DNA through ion pumps [36]. The migration of DNA solution in the *i.d.* layer by passing the time after the first D_EP_ application might occur. This may be related to the plasmid concentration at the administration site. Wolf et al. [37] reported that higher plasmid concentration increased the proportion of positively transfected cells but decreased cell viability. Therefore, the aforementioned mechanism might be involved in higher luciferase activity with repeated *D*_EP_ application at 5 min intervals, but further investigation is needed to elucidate the reason.

Sugibayashi et al. [38] reported that the distribution of the electric field should be considered in addition to the application voltage to optimize the effect of EP on the transdermal delivery of drugs. They used parallel-type electrodes in addition to needle-type electrodes, and parallel electrodes gave an almost even electric field, but there was a lower distribution in the electric field intensity for needle-type electrodes. The electrode used in the present study was placed parallel. Thus, higher luciferase expression might have been observed even in *D*_EP_ application with 20 V.

The skin after repeated *D*_EP_ application with 20 V exhibited similar thickness compared with non-treated skin. In the current study, no experiments were conducted for acute or chronic toxicities after single *D*_EP_ and repeated applications. Further evaluation for these factors should be conducted to show the possibility of the usefulness of *D*_EP_ for enhancement of pDNA uptake into skin tissue.

As research progresses, electroporation with a depot-type electrode system may offer advantages in clinical settings, particularly for treatments requiring repeated EP sessions. Implanted electrodes can reduce tissue damage and patient discomfort by eliminating repeated electrode insertions into the body, potentially improving adherence to long-term therapeutic regimens. Direct contact with dermal tissue may allow for effective EP at lower voltages, further minimizing pain and tissue damage. This approach could benefit DNA vaccines and gene therapies requiring prolonged or intermittent expression, as it may enable more consistent and higher levels of gene expression over time.

## 5. Conclusions

This study confirmed that applying a voltage by *i.d.* administration directly achieved high protein expression levels even at low voltages. In addition, repeated *D*_EP_ application further enhanced gene transfection efficiency. This approach provided increased gene expression levels compared with conventional plate-type EP methods. However, this study only evaluated EGFP and luciferase as model proteins, and no evaluation of functional activity was conducted. In addition, further investigation should be conducted to maximize gene expression efficiency with *D*_EP_ parameters such as the voltage, number of pulse applications, duration of pulse application, electrode design, and electrode array layout.

This study emulates conditions with electrodes embedded within the dermis, directly applying a voltage to the dermal layer, thus bypassing the electrical resistance characteristic of the SC surface. Comparative analyses indicated that this methodology results in significantly higher protein expression levels than *C*_EP_. These findings suggest that direct *D*_EP_ may enhance the efficiency of transdermal drug delivery. This method is an invasively applied EP with an implanted device. Therefore, a conventional EP that applies voltage to the skin surface would be simpler and more practical due to its ease of application. This study was conducted in the belief that with the future development of implantable devices, a variety of drug delivery system applications will be investigated. Further study is needed, but this result may be one of the potential results for further drug delivery system development with implanted devices.

## Data Availability

The study data are contained within the article.

## Abbreviation

ANOVA	one-way analysis of variance
*C*_EP_	conventional stratum corneum electroporation
CLSM	confocal laser scanning microscope
*D*_EP_	direct electroporation at an intradermal site
EGFP	enhanced green fluorescent protein
EP	electroporation
GFP	green fluorescent protein
H.E.	hematoxylin and eosin
hMN	hollow-type microneedle
*i.d.*	intradermal
IVIS	in vivo imaging system
MN	microneedle
PBS	phosphate-buffered saline
pDNA	plasmid DNA
RLU	relative light units
ROI	region of interest
SC	stratum corneum
